# CD86 and IL-12p70 Are Key Players for T Helper 1 Polarization and Natural Killer Cell Activation by Toll-Like Receptor-Induced Dendritic Cells

**DOI:** 10.1371/journal.pone.0044266

**Published:** 2012-09-04

**Authors:** Felix S. Lichtenegger, Katharina Mueller, Bettina Otte, Barbara Beck, Wolfgang Hiddemann, Dolores J. Schendel, Marion Subklewe

**Affiliations:** 1 Department of Internal Medicine III, Klinikum der Universität München, Munich, Germany; 2 Institute of Molecular Immunology, Helmholtz Zentrum München, German Research Center for Environmental Health, Munich, Germany; University of Bergen, Norway

## Abstract

**Background:**

Dendritic cells (DCs) determine the activation and polarization of T cells via expression of costimulatory molecules and secretion of cytokines. The function of DCs derived from monocytes *ex vivo* strongly depends on the composition of the maturation cocktail used.

**Methodology/Principal Findings:**

We analyzed the effect of costimulatory molecule expression and cytokine secretion by DCs on T and natural killer (NK) cell activation by conducting a head-to-head comparison of a Toll-like receptor (TLR) agonist-based cocktail with the standard combination of proinflammatory cytokines or IL-10 alone. We could show that TLR-induced DCs are characterized by a predominance of costimulatory over coinhibitory molecules and by high secretion of IL-12p70, but not IL-10. Functionally, these signals translated into an increase in IFN-γ secreting Th1 cells and a decrease in regulatory T cells. T cell activation and polarization were dependent on IL-12p70 and CD86, but remarkably not on CD80 signaling. By means of IL-12p70 secretion, only TLR-induced DCs activated NK cells.

**Conclusions/Significance:**

TLR-matured DCs are highly suitable for application in immunotherapeutic strategies that rely on strong type 1 polarization and NK cell activation. Their effects particularly depend on high CD86 expression and IL-12p70 secretion.

## Introduction

First identified and isolated in 1973 [1], dendritic cells (DCs) have since evolved in our understanding from mere “accessory” cells to essential initiators and modulators of innate and adaptive immune responses. Acting as professional antigen-presenting cells, they effectively stimulate naïve and memory T cells [2]. Due to their high potency to induce tumor-specific T cells [3]–[5], DCs have been used in cancer immunotherapy for 17 years [6]. Although antigen-specific immune responses were elicited in the majority of patients, clinical effects have been limited [7], [8]. However, with new knowledge of DC biology rapidly increasing, several impediments are now better understood and can be overcome in the design of future studies [9]–[12].

The vast majority of DC preparations used for vaccination studies have been generated from autologous peripheral blood monocytes, using a two-step process. First, monocytes are differentiated into immature DCs by culturing them with IL-4 and GM-CSF. Culture time has traditionally been 5 to 6 days, but it has been shown that 24 hours are sufficient [13]. Subsequently, those cells are matured by addition of various cytokines and other additives for 24 to 48 hours.

The type and concentration of these substances is decisive for the characteristics of the resultant DCs. As recently reviewed [14], many different maturation cocktails have been utilized for DC generation. The gold standard so far has been the combination of TNF-α, IL-1β, IL-6 and PGE_2_ [15]. This protocol was designed to enhance maturation markers, migratory and immunostimulatory properties of DCs and has been used, with minor variations, for most clinical studies applying DCs for immunotherapy to date [16]–[19].

The specific binding of a peptide-loaded major histocompatibility complex molecule to a T cell receptor is the major signal for activation and differentiation of T cells (signal 1). However, the extent and type of the resulting T cell response is determined by the interaction of costimulatory molecules on antigen-presenting cells with the respective ligands on T cells (signal 2) and the secretion of cytokines (signal 3) [20]. The T cell response is thus substantially influenced by the characteristics of the stimulating DCs [21]–[23], and the analysis of these signals provides a better understanding of the stimulatory capacities of a DC population [21], [24].

IL-12p70 is of special importance for Th1 polarization [25], resulting in the type of immune response that is essential for an effective reaction against cancer and cellular pathogens. However, bioactive IL-12p70 is not produced by DCs matured with the combination of TNF-α, IL-1β, IL-6 and PGE_2_. Therefore, alternative ways of DC generation have been analyzed. When it was discovered that Toll-like receptor (TLR) agonists induce DCs with Th1-polarizing capacity [26], [27], these agents were increasingly included in maturation mixtures, especially the TLR3 ligand polyI:C [28] and the TLR7/8 agonist R848 [29], [30]. The combination of proinflammatory cytokines including IFN-γ with PGE_2_ and the TLR ligands polyI:C and R848 applied to immature DCs after two days of differentiation time resulted in DCs that actively secreted IL-12p70 [31], [32].

The expression profile of costimulatory molecules has not been determined and compared in great detail in differently matured DC populations so far. Of course, it has long been known that mature DCs express the costimulatory molecules CD80 (B7-1) and CD86 (B7-2) and that their engagement influences the direction of T cell differentiation [33]–[35]. However, since then two sets of costimulatory molecules were elucidated, the B7 family [36]–[39] that includes CD80 and CD86, and the family of TNF receptors and their ligands [40]. Differential effects of these molecules on phenotype and function of stimulated T cells were discovered. However, little to nothing is known about the influence of different maturation protocols on the expression of these molecules on DCs.

Functional effects of DCs on T cells have traditionally been probed by proliferation of allogeneic T cells. This is a rather crude assay because HLA differences will cause T cells to proliferate largely independently of additional stimulatory signals. In contrast, an assay testing T cell stimulation in an autologous system free of exogenous cytokines or allogeneic stimulation is more relevant to the physiologic situation as well as therapeutic manipulations given that translational applications of DCs generally use autologous cells [41]. In such a setting, more subtle evidence of the different T cell subsets induced by DCs can be acquired, differentiating between the induction of activated and regulatory T cells and analyzing their polarization. Phenotypic markers for human regulatory T cells do not identify distinct populations as they exist in mice [42]. However, the combination of FoxP3 expression and absent or low-level surface expression of the IL-7 receptor α (CD127) is helpful to identify human regulatory T cells [43], [44]. Successful clinical application of DCs depends on their capability to induce immune responses in an autologous setting. Specifically, a strong Th1 response and natural killer (NK) cell activation are integral parts of immunotherapeutic strategies relying on DCs [19], [45], [46].

Thus, the goal of this study was to analyze different DC subpopulations generated side-by-side from the same blood donors for a comprehensive panel of costimulatory molecules, their cytokine secretion patterns and the resulting functional impacts of these signals on the activation of T and NK cells. Particularly, a TLR ligand-including cocktail recently developed for future application in clinical studies [31], [32] was compared to the traditionally used cocktail of proinflammatory cytokines. In most experiments, a subset of DCs matured with IL-10 was added as a control for lack of immunostimulation, based on previous reports that addition of IL-10 leads to a type of DCs with more immunosuppressive characteristics [47], [48]. We were specifically interested in analyzing the significance of individual costimulatory or cytokine signals on the stimulatory capability of the DCs. The data presented here elucidate why TLR-matured DCs are superior T and NK cell activators and are thus highly suitable for the development of immunotherapeutic strategies based on DC vaccination.

## Results

### TLR-3-DCs Have a Prominent Positive Costimulatory Profile, While cc-7-DCs Express More Coinhibitory Molecules

**Figure 1:**
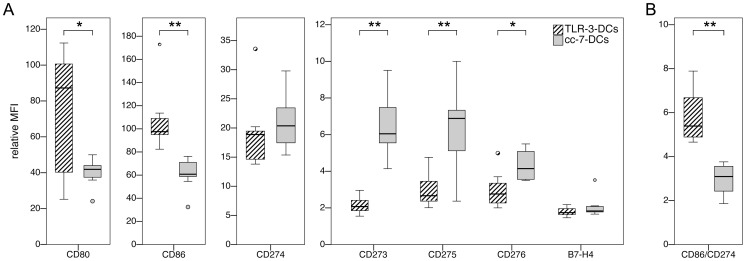
Costimulatory profiles of TLR-3-DCs and cc-7-DCs. TLR-3-DCs and cc-7-DCs were generated from peripheral blood of healthy donors, and expression of various costimulatory markers was analyzed by flow cytometry. *, p<0.05; **, p<0.01. (A) Expression of the cell surface antigens on both DC populations (n = 7 for B7-H4, n = 8 for CD276, n = 10 for all other markers). (B) Comparison of the CD86/CD274 ratio for TLR-3-DCs and cc-7-DCs (n = 10).

Monocytes were obtained from peripheral blood of 10 healthy donors. Two populations of DCs were generated in side-by-side assays, using the conventional maturation cocktail with a 7-day culture period (cc-7-DCs) versus a TLR agonist-based maturation cocktail and a 3-day culture period (TLR-3-DCs), as specified in Materials and Methods. Analysis of basic surface markers (CD14, CD83, HLA-DR, CCR-7) showed that mature DCs were generated by both protocols (data not shown). However, the level of CD40 expression on TLR-3-DCs, reflected by the relative mean fluorescence intensity (MFI), was more than double that of cc-7-DCs (p = 0.005; data not shown). We extended the phenotype analysis of both DC populations to include a broad set of B7 molecules (CD80 = B7-1; CD86 = B7-2; CD273 = PD-L2; CD274 = PD-L1; CD275 = B7-H2 = ICOS ligand; CD276 = B7-H3; B7-H4; Fig. 1A). TLR-3-DCs were found to express high amounts of CD80 and CD86, with median relative MFIs of 87.2 and 97.6, respectively. The expression of the other markers was lower, with relative MFIs ranging from 1.74 for B7-H4 to 18.9 for CD274. In comparison to TLR-3-DCs, the levels of CD80 and CD86 on cc-7-DCs were much lower (MFIs of 41.9 and 60.8, respectively; p = 0.013 for CD80 and p = 0.005 for CD86). All other markers showed a similar (CD274; B7-H4) or significantly higher (CD273; CD275; CD276) expression on cc-7-DCs. The ratio of CD86 and CD274 expression was calculated for each preparation as an indication of positive costimulatory capacity (Fig. 1B). The values of this ratio were far higher for TLR-3-DCs than for cc-7-DCs (5.4 vs. 3.1; p = 0.005).

### Shortening of Culture Time Results in Convergence of the Costimulatory Expression Profile

**Figure 2:**
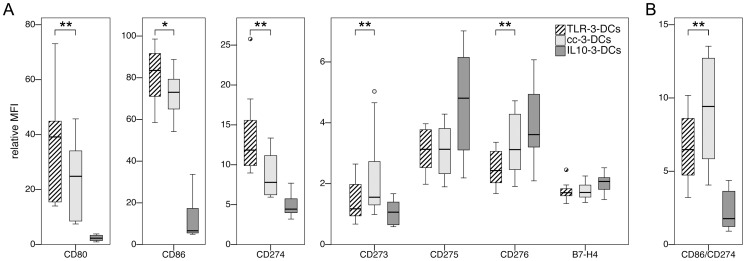
Costimulatory profiles of TLR-3-DCs, cc-3-DCs and IL10-3-DCs. TLR-3-DCs, cc-3-DCs and IL10-3-DCs were generated from peripheral blood of healthy donors, and expression of various costimulatory markers was analyzed by flow cytometry. Differences between TLR-3-DCs and cc-3-DCs were tested. *, p<0.05; **, p<0.01. (A) Expression of the cell surface antigens on all three DC populations (n = 10). (B) Comparison of the CD86/CD274 ratio for all three DC populations (n = 10).

Monocytes were obtained from peripheral blood of 10 healthy donors, and three populations of DCs were generated in side-by-side assays, using the TLR agonist-based maturation cocktail (TLR-3-DCs), the conventional maturation cocktail (cc-3-DCs) or IL-10 alone for maturation (IL10-3-DCs), each with a 3-day culture period. The DC phenotype was analyzed by flow cytometry (Fig. 2). Expression of the positive costimulatory molecules CD80 and CD86 measured by median relative MFI was lower for cc-3-DCs compared to TLR-3-DCs in the majority of the donors (24.8 vs. 39.1 for CD80, p = 0.005; 73.0 vs. 83.5 for CD86, p = 0.013). However, median differences between expression levels of cc-3-DCs and TLR-3-DCs were significantly lower (8.60 for CD80 and 8.59 for CD86) compared to differences between cc-7-DCs and TLR-3-DCs (44.17 and 36.21, respectively). Similarly, expression of the coinhibitory molecules converged, as seen for CD273 (median difference of 4.1 between cc-7-DCs and TLR-3-DCs; only 0.5 between cc-3-DCs and TLR-3-DCs) and CD275 (median difference of 3.9 between cc-7-DCs and TLR-3-DCs; only 0.1 between cc-3-DCs and TLR-3-DCs). IL10-3-DCs exhibited a profoundly different phenotype, with higher CD14 expression (data not shown) and very low expression of CD80 and CD86.

### TLR-3-DCs Secret IL-12p70, in Contrast to cc-3-DCs and IL10-3-DCs

**Figure 3:**
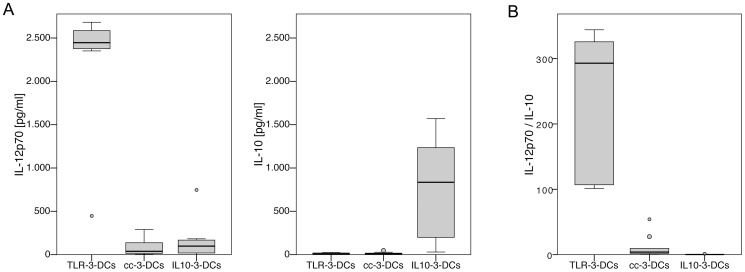
Cytokine secretion patterns of TLR-3-DCs, cc-3-DCs and IL10-3-DCs. DCs generated from peripheral blood of healthy donors were analyzed for their cytokine secretion patterns (n = 9). (A) Mature DCs were cocultured with CD40L expressing mouse fibroblasts for 24 hours, the concentration of IL-12p70 and IL-10 in the supernatants was measured by CBA, and the difference to the basal secretion of the same cell populations without CD40 ligation was calculated. (B) Comparison of the IL-12p70/IL-10 ratio for TLR-3-DCs, cc-3-DCs and IL10-3-DCs.

DCs generated from 9 healthy donors were cocultured with a murine fibroblast cell line (L-929) stably transfected with CD40 ligand. Supernatants of 24-hour cocultures were analyzed for secreted levels of IL-12p70 and IL-10 by cytometric bead array (CBA); cocultures with non-transfected L-929 cells were used as a background control (Fig. 3). IL-12p70 was secreted in large amounts (median near 2.5×10^3^ pg/ml) by TLR-3-DCs, while cc-3-DCs hardly produced measurable amounts of this cytokine (median 38 pg/ml, p = 0.008; Fig. 3A). Production of IL-10 by TLR-3-DCs as well as cc-3-DCs was just above detection limit (median of 8 pg/ml each; Fig. 3A). The ratio of IL-12p70 and IL-10 production is shown in Fig. 3B. Its median was 293 for TLR-3-DCs and 4 for cc-3-DCs (p = 0.008). Cytokine secretion of cc-7-DCs was very similar to cc-3-DCs (Fig. S1). IL10-3-DCs secreted high amounts of IL-10 (median of 835 pg/ml) and very small amounts of IL-12p70 (median of 98 pg/ml), resulting in a median IL-12p70 to IL-10 ratio of 0.14 (Fig. 3A and B).

### TLR-3-DCs Preferentially Induce Activated IFN-γ Secreting Th1 Cells

**Figure 4:**
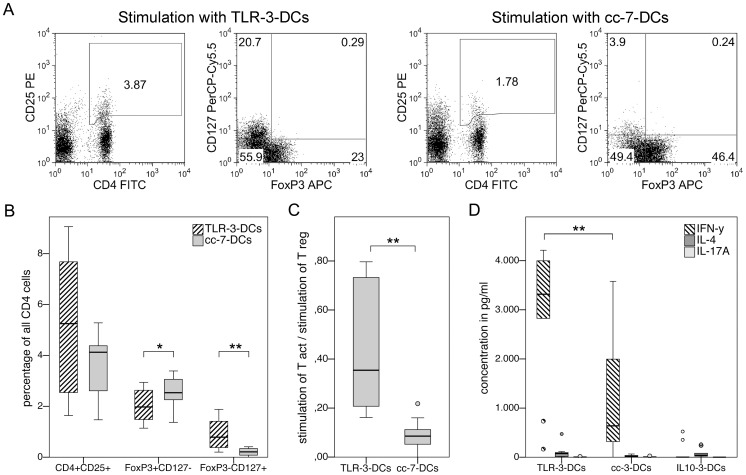
Preferential induction of activated, IFN-γ secreting T cells by TLR-3-DCs. (A to C) TLR-3-DCs and cc-7-DCs generated from peripheral blood of healthy donors were cocultured with autologous monocyte-depleted (non-adherent) PBMCs for 24 hours, and antigen-independent stimulatory capacity of the DCs on regulatory and activated T cells was quantified using flow cytometry with the markers CD4, CD25, CD127 and FoxP3. *, p<0.05; **, p<0.01. (A) Data from one representative donor. Gating strategies used to determine different T cell subsets. (B) Capacity of TLR-3-DCs and cc-7-DCs to stimulate CD4^+^CD25^+^ T cells and their regulatory (FoxP3^+^CD127^-^) and non-regulatory activated (FoxP3^-^CD127^+^) subsets. Differences between stimulated and unstimulated cells are shown (n = 10). (C) Ratio of activated and regulatory T cells induced by coculture. (D) TLR-3-DCs, cc-3-DCs and IL10-3-DCs were cocultured with CD3-selected autologous T cells for 4 days. Supernatants were analyzed for secretion of IFN-γ, IL-4 and IL-17A by CBA. Differences between stimulated and unstimulated cells are shown (n = 10).

DCs generated from 10 healthy donors were cocultured with autologous monocyte-depleted (non-adherent) peripheral blood mononuclear cells (PBMCs) for 24 hours, and the antigen-independent stimulating capacity for regulatory and non-regulatory activated T cells was quantified using surface staining for CD4, CD25 and CD127 as well as intracellular staining for FoxP3. Fig. 4A shows the gating strategies used to determine the populations of interest for one representative donor. Percentages of the different T cell subsets in relation to all CD4^+^ cells are plotted in Fig. 4B after subtraction of the respective unstimulated background control. When total CD4^+^CD25^+^ cells were compared, there was a trend toward higher stimulation by TLR-3-DCs, which came close to statistical significance (5.3% vs. 4.1%; p = 0.09). CD127 and FoxP3 were used to further subdivide this population. CD4^+^CD25^+^FoxP3^+^CD127^−^ cells represent regulatory T cells, while the combination CD4^+^CD25^+^FoxP3^−^CD127^+^ was selected to represent activated non-regulatory T cells. Significantly more regulatory cells were induced by cc-7-DCs than by TLR-3-DCs (2.5% vs. 2.0%; p = 0.037). In contrast, numbers of activated non-regulatory T cells were reciprocally impacted: more CD4^+^CD25^+^FoxP3^−^CD127^+^ cells were induced by stimulation with TLR-3-DCs (0.8%), with a highly significant difference to cc-7-DCs (0.2%; p = 0.007). The ratio of activated to regulatory T cells following these definitions differed significantly between both populations: the median ratio was 0.35 for TLR-3-DCs and 0.09 for cc-7-DCs (p = 0.005; Fig. 4C).

In a different set of experiments, DCs generated from 10 healthy donors were cocultured with CD3-selected autologous T cells for 4 days, and supernatants were analyzed for secretion of IFN-γ, IL-4 and IL-17A by CBA to determine T cell polarization (Fig. 4D). Most prominently, TLR-3-DCs caused very high IFN-γ secretion, with a median of 3318 pg/ml. The coculture with cc-3-DCs resulted in lower IFN-γ secretion (median of 638 pg/ml; p = 0.007 for comparison with TLR-3-DCs), while IL10-3-DCs did not induce IFN-γ secretion at all (median of 1.3 pg/ml). IL-4 and IL-17A concentrations were found to be just above the detection limit in the supernatants of all of the cocultures.

### Th1 Polarization Capacity of TLR-3-DCs is Dependent on CD86 Signaling and IL-12p70

**Figure 5:**
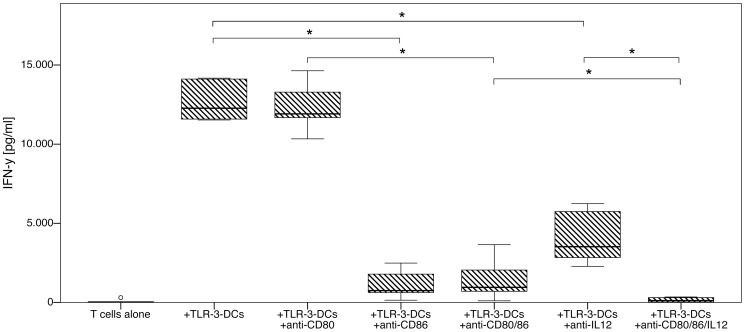
Effect of costimulation and IL-12p70 blockade on Th1 polarization by TLR-3-DCs. TLR-3-DCs generated from peripheral blood of 6 healthy donors were cocultured with CD3-selected autologous T cells for 4 days either alone or with addition of various combinations of blocking antibodies. Supernatants were analyzed for secretion of IFN-γ by ELISA. T cells without DC stimulation were used as a negative control. Box-and-whisker plots for the different conditions are shown. *, p<0.05.

TLR-3-DCs generated from 6 healthy donors were cocultured with CD3-selected autologous T cells for 4 days, and supernatants were analyzed for secretion of IFN-γ by ELISA. During the coculture, various combinations of antibodies with the capacity to neutralize the respective signaling pathway were added (Fig. 5). CD80 signaling did not effect Th1 polarization, as its blockade did not reduce IFN-γ secretion compared to TLR-3-DCs alone (median of 11912 pg/ml with anti-CD80, 12270 pg/ml without anti-CD80) or to TLR-3-DCs with anti-CD86 (median of 954 pg/ml with additional anti-CD80, 747 pg/ml without anti-CD80). In contrast to CD80, the blockade of CD86 reduced the capacity of T cells to secrete IFN-γ by a factor of approximately 10 compared to TLR-3-DCs alone (median of 747 pg/ml with anti-CD86, 12270 pg/ml without anti-CD86; p = 0.028) or to TLR-3-DCs with anti-CD80 (median of 954 pg/ml with additional anti-CD86, 11912 pg/ml without anti-CD86; p = 0.028). In a similar manner, the blockade of IL-12p70 strongly reduced IFN-γ secretion compared to TLR-3-DCs alone (median of 3522 pg/ml with anti-IL12, 12270 pg/ml without anti-IL12; p = 0.028). Importantly, the blockade of IL-12p70 had a synergistic effect to the blockade of CD80 and CD86 costimulation (median of 118 pg/ml with additional anti-IL12 and 954 pg/ml without anti-IL12; p = 0.028), and the blockade of costimulation had a synergistic effect to the blockade of IL-12p70 (median of 118 pg/ml with additional anti-CD80 and anti-CD86 and 3522 pg/ml without these antibodies; p = 0.028).

### NK Cell Activation by TLR-3-DCs Depends on Secretion of IL-12p70

**Figure 6:**
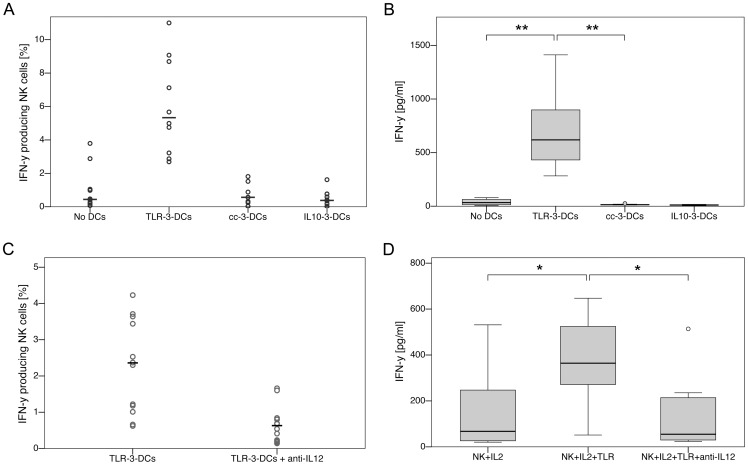
IL-12p70 dependency of NK cell activation by TLR-3-DCs. (A/B) DCs generated from peripheral blood of 10 healthy donors were cocultured with autologous monocyte-depleted (non-adherent) PBMCs for 24 hours with addition of IL-2. Activation of NK cells was analyzed by intracellular IFN-γ staining of CD3^−^CD56^+^ cells (A; circles represent single experiments, the mean is displayed as horizontal line) and by ELISA measurement of IFN-γ in the supernatant (B; box-and-whisker plots). (C/D) In a similar set of experiments, cocultures of TLR-3-DCs and autologous monocyte-depleted PBMCs were compared with or without addition of IL-12p70 blocking antibody. Activation of NK cells was analyzed by intracellular IFN-γ staining of CD3^−^CD56^+^ cells (C; n = 13) and by ELISA measurement of IFN-γ in the supernatant (D; n = 8). *, p<0.05; **, p<0.01.

DCs generated from 10 healthy donors were cocultured with autologous monocyte-depleted (non-adherent) PBMCs for 24 hours with addition of IL-2, and NK cell activation was measured by intracellular IFN-γ staining of CD3^−^CD56^+^ cells (Fig. 6A) and by ELISA measurement of IFN-γ in the supernatant (Fig. 6B). We found that TLR-3-DCs were capable of inducing IFN-γ secretion by NK cells, while cc-3-DCs and IL10-3-DCs were not (p = 0.005 for differences between TLR-3-DCs and IL-2 alone or cc-3-DCs). Addition of an IL-12 blocking antibody to the coculture with TLR-3-DCs abolished this effect: both intracellular staining (Fig. 6C) and measurement in the supernatant (Fig. 6D) showed that IFN-γ levels were reduced to background when the signaling of IL-12p70 was blocked.

## Discussion

It has long been known that upregulation of the costimulatory molecules CD80 and CD86 is a crucial step in DC maturation [49]. Signals resulting from the interaction of these molecules with CD28 on responding lymphocytes are critical for initial cell cycle progression, IL-2 production and clonal expansion. The role of other B7 family members is ambiguous with different, albeit overlapping, functions concerning priming, proliferation and maturation of effector cells [39], [40]. Nevertheless they all display, partially or predominantly, coinhibitory functions, contributing to regulation of the immune response. Although the expression levels of most of the molecules analyzed here varied considerably between blood donors, the side-by-side comparison of multiple donors allowed us to demonstrate that TLR-3-DCs are characterized by a prominent positive costimulatory profile, while cc-7-DCs express higher levels of the coinhibitory molecules within the B7 family. A direct comparison between cc-7-DCs and cc-3-DCs was not the focus of our analysis. However, the data presented here allow to speculate that the shortening of culture time alone results in enhanced expression of costimulatory molecules in cc-3-DCs, resulting in a phenotype much more similar to TLR-3-DCs than that of cc-7-DCs. For the evaluation of DC application in anticancer immunotherapy, it is important to consider that the majority of clinical vaccination trials carried out so far was based on DC populations similar to cc-7-DCs [16]–[19]. Unfavorable costimulatory profiles could therefore have contributed to the partially unsuccessful clinical results.

Besides costimulation, the cytokines secreted by DCs after T cell contact are of utmost importance for their effects on adaptive immunity and their applicability in immunotherapy. All DC populations were therefore further characterized by analyzing their cytokine secretion patterns following CD40 ligation as a mimic for encounter with T cells. IL-12p70 is an essential component of the cytokine milieu in the case of Th1 polarization [25], [50], while IL-10 favors induction of tolerance. Recent studies with *in vitro* generated DCs have argued in favor of using DC populations with a high capacity to secrete IL-12p70 [28], [29], [31], [51]. In the majority of these publications, IL-12p70 was measured in the supernatant of DC maturation culture, with ranges reported up to several ten thousands of pg/ml [29]. Considering the use of DCs *in vivo*, it is more relevant to analyze IL-12p70 secretion upon interaction of DCs with T cells as simulated in the signal-3 assay. Cytokine concentrations measured this way are usually considerably lower. Dohnal et al. defined an IL-12p70 concentration of 100 pg/ml as release criterion for their cancer vaccine [51], but the required amounts for Th1 polarization and clinical efficacy *in vivo* are not known. As previously described, cc-7-DCs and cc-3-DCs produced very small amounts of IL-12p70 in this *in vitro* study. In contrast, TLR-3-DCs secreted large amounts of this cytokine, far above 100 pg/ml for every single donor. IL-10 production was much lower and almost negligible. The calculation of the ratio of IL-12p70 to IL-10 secretion as an index for the Th1-polarizing capacity of a given population of mature DCs demonstrated that TLR-3-DCs are decidedly superior to cc-7-DCs and cc-3-DCs in secreting cytokines that favor a Th1 response polarization.

The stimulatory capacity of different DC populations on autologous CD4 T cells was analyzed with respect to induction of regulatory versus activated T cells. While there was no statistical significant difference in stimulation of total CD4^+^C25^+^ cells, the subdivision of this inhomogeneous population by expression of CD127 and FoxP3 revealed that conventionally generated DCs mainly induce regulatory T cells, while the ratio is shifted toward activated non-regulatory T cells after interaction with TLR-matured DCs. The clinical significance of this subdivision of CD4^+^CD25^+^ cells was highlighted in an immunotherapy study with malignant melanoma patients [17]: the application of an anti-CD25 antibody proved to be too unspecific to only delete T regulatory cells, since it also eliminated effector cells, leading to reduced instead of enhanced immune responses. Therefore, it is of great importance for potential application of DCs in immunotherapy to investigate such differential effects on the activation of autologous T cells. In spite of the high natural variability among blood donors, the difference between TLR-3-DCs and cc-7-DCs proved to be highly significant in our experiments.

When further characterizing the T cell activation capacity of the DC subsets with respect to polarization of Th1, Th2 or Th17 cells, we found that cocultivation with TLR-3-DCs caused a very high IFN-γ secretion. As IL-12p70 is known to be a very potent stimulator of Th1 polarization, this effect was to be expected. However, cc-3-DCs still induced a considerable IFN-γ secretion, although they did not produce any IL-12p70. Considering that the costimulatory profile of cc-3-DCs is highly positive and almost similar to TLR-3-DCs, we analyzed the significance of positive costimulation as well as IL-12p70 secretion for Th1 polarization by TLR-3-DCs. Surprisingly, we found that CD80 signaling did not effect Th1 polarization at all, while CD86 and IL-12p70 acted synergistically to induce IFN-γ secretion. For cc-3-DCs, we conclusively found that blockade of CD86 almost completely abolished the partial Th1 polarization, while CD80 blockade did not have an effect on IFN-γ levels (data not shown). This finding is in contrast to the more widespread notion that CD86 rather induces type 2 polarization of naïve T cells, whereas CD80 is a more neutral differentiation signal or rather induces type 1 polarization [34], [35].

Besides Th1 polarization, another functional effect of high importance for the potential application of TLR-3-DCs in immunotherapy is their activation of NK cells [19], [45], [46]. In accordance with the textbook knowledge that IL-12p70 is instrumental for NK cell activation, only TLR-3-DCs were capable of inducing IFN-γ secretion by NK cells, and addition of an IL-12 blocking antibody to the coculture abolished this effect. Contrary to Th1 polarization, there was no partial effect left after blockade.

In conclusion, our studies showed that DCs generated using the specific TLR-based maturation cocktail established in our lab were characterized by a predominance of costimulatory over coinhibitory molecules and high IL-12p70, but no IL-10 secretion. When compared to DCs matured by the standard combination of proinflammatory cytokines, this resulted in an increase in activated T cells and a decrease in regulatory T cells within stimulated autologous T cell populations. The activated T cells proved to be IFN-γ secreting Th1 cells. This stimulation was shown to be dependent on IL-12p70 secretion as well as CD86 signaling, but not CD80 signaling. The high CD86 expression and IL-12p70 secretion seen in TLR-3-DCs thus enabled them to induce a strong Th1 immune response. NK cells were also activated by this DC subset entirely dependent on IL-12p70 secretion. We conclude that DCs matured with this TLR agonist-based cocktail are highly suitable for application in those immunotherapeutic strategies that rely on a strong type 1 polarization and NK cell activation, especially in cancer immunotherapy.

## Materials and Methods

### Media and Reagents

Very low endotoxin RPMI 1640 medium (Biochrom, Berlin, Germany) supplemented with 1.5% human serum (serum pool of AB positive adult males; Institute for Transfusion Medicine, Suhl, Germany) – hereafter named DC medium – was used for the generation of DCs and all coculture experiments. Freezing medium consisted of 90% FCS (PAN Biotech GmbH, Aidenbach, Germany) and 10% DMSO (SERVA Electrophoresis, Heidelberg, Germany). The following reagents were used to generate DCs: GM-CSF (Leukine; Bayer HealthCare, Seattle, WA, USA), rhIL-4, rhIL-1β, rhIL-6, rhIL-10, TNF-α (all Immunotools, Friesoythe, Germany), PGE_2_ (Prostin E2; Pharmacia Ltd, Kent, UK), IFN-γ (Imukin; Boehringer Ingelheim Pharma, Ingelheim am Rhein, Germany), polyI:C and R848 (both InvivoGen, San Diego, CA, USA).

### Cell Isolation and Generation of DCs

After written informed consent, peripheral blood samples were collected from healthy donors under a clinical protocol entitled “In vitro studies to establish new immunotherapies for acute myeloid leukemia and other hematological neoplasias”. Both the consent form and the protocol were approved by the institutional review board (Ethikkommission bei der LMU München). PBMCs were isolated by standard density gradient centrifugation (Biocoll; Biochrom, Berlin, Germany). Monocytes were isolated by plastic adherence in 6-well plates (BD, Franklin Lakes, NJ, USA) at a concentration of 1–2×10^7^ PBMCs/well in 3 ml DC medium. Non-adherent cells were kept at 37°C and 5% CO_2_ until further use three days later. Only for the experiments shown in Fig. 4 A to C, cells were temporarily cryopreserved. Viability after thawing was always above 90%. Monocytes were cultured for 7 days (cc-7-DCs) or 3 days (all other DC types) at 37°C and 5% CO_2_ in DC medium supplemented with 800 IU/ml GM-CSF and 580 IU/ml IL-4, added freshly every other day. Maturation of DCs was achieved by addition of the following factors during the last 24 hours of culture time: IL-1β (2000 IU/ml), PGE_2_ (250 ng/ml), TNF-α (1100 IU/ml), IFN-γ (5000 IU/ml), polyI:C (20 ng/ml) and R848 (1 µg/ml) for TLR-3-DCs; IL-1β (2000 IU/ml), IL-6 (1100 IU/ml), PGE_2_ (1000 ng/ml) and TNF-α (1100 IU/ml) for cc-7-DCs and cc-3-DCs; IL-10 (40 ng/ml) for IL10-3-DCs. Mature DCs were harvested, washed twice and used directly for the various assays. Existence of residual amounts of IFN-γ was ruled out by ELISA (data not shown). The purity of the DC preparations was around 80% as shown in Fig. S2. All phenotypical data shown for DCs (Figs.1 and 2) were analyzed after gating on the DC population as shown in Fig. S2.

### Surface Phenotyping of DCs

Immunofluorescent staining of cell surface antigens was performed using a panel of fluorescence conjugated monoclonal antibodies: CD273 (PE, MIH18), CD275 (PE, MIH12; both eBioscience, San Diego, CA, USA), CD80 (PE, L307.4), CD86 (FITC, 2331 (FUN-1)), CD274 (FITC, MIH1; all BD Biosciences, San Jose, California, USA), CD276 (FITC, MIH42) and B7-H4 (FITC, MIH43; both AbD Serotec, Oxford, UK). Corresponding isotype controls were used (eBioscience). Cells were analyzed using a FACSCalibur (BD Biosciences). Post-acquisition data analysis was performed with FlowJo 8 software (Tree Star, Ashland, OR, USA). The relative MFI was calculated by dividing the MFI of the measured population by the MFI of cells stained with the isotype-matched antibody.

### Signal 3 Assay of Cytokine Secretion following CD40 Ligation

Secretion of IL-12p70 and IL-10 by DCs was tested in a coculture with CD40 L-expressing mouse fibroblasts as a mimic for interactions with activated T cells, as described before [32]. Briefly, DCs were cocultured for 24 hours with irradiated L-929 cells (permanent cell line CCL-1; American Type Culture Collection ATCC), stably transfected with human CD40 L, and supernatants were analyzed by CBA. Non-transfected L-929 cells were used as a background control.

### Measurement of Autologous T Cell Stimulation and Polarization Capacity

Non-adherent PBMCs were thawed and plated in 12-well plates (BD Biosciences) at a concentration of 1×10^6^ cells/well in 1 ml of DC medium together with 1×10^5^ DCs for 24 hours. As a background control, non-adherent PBMCs were cultured without DCs. To quantify activated and regulatory T cells, cell surface antigens were stained using the following monoclonal antibodies: CD4 (FITC, VIT4), CD25 (PE, 4E3; both Miltenyi Biotec, Bergisch-Gladbach, Germany), and CD127 (PerCP-Cy5.5, eBioRDR5; eBioscience). Intracellular FoxP3 staining was performed according to the manufacturer’s instructions (APC, 3G3; Miltenyi Biotec). In order to set the correct gates, appropriate isotype controls were used, and in the case of FoxP3 a fluorescence-minus-one control with addition of an intracellular isotype. The stimulation actually achieved by the coculture with DCs was determined by subtracting the percentage of the respective population measured within the unstimulated PBMCs. To test the polarization capacity of the DCs for T cells, non-adherent PBMCs were selected for CD3 positivity by MACS® Cell Separation (Miltenyi Biotec), according to the manufacturer’s instructions. T cells were cocultured for 4 days in 96-well plates (BD Biosciences) at a concentration of 2×10^5^ cells/well together with 3×10^4^ DCs in 200 µl DC medium. Supernatants of 3 replicate wells were pooled, and secreted cytokines were analyzed by ELISA or CBA. T cells without DC stimulation were used as a negative control. In order to prevent any transfer of residual cytokines from DC maturation into the coculture experiments, all DC preparations were washed twice before adding them to the CD3 cells. Exemplarily, for two of the donors the complete absence of IFN-γ at the start of the coculture was verified by ELISA (data not shown). In some experiments, various antibodies were added during coculture to block specific signaling pathways: anti-human CD80 (2D10), anti-human CD86 (IT2.2; both BioLegend, San Diego, California, USA) and anti-human IL-12/IL-23 p40/70 (C8.6, eBioscience), each of them at a concentration of 10 µg/ml.

### Measurement of NK Cell Activation

3×10^4^ DCs were cocultured with 3×10^5^ autologous non-adherent PBMCs/well in 96-well plates with addition of IL-2 (Proleukin S, Novartis International AG, Basel, Switzerland) at 1000 IU/ml. After incubation for 20 hours, Golgi stop solution consisting of Monensin at 25 µM and Brefeldin A at 10 µg/ml (both Sigma-Aldrich, St. Louis, MO, USA) were added for additional 4 hours. Supernatants were collected and analyzed for IFN-γ secretion by ELISA. Additionally, the cells were harvested, surface stained for CD3 (PerCP/Cy5.5, HIT3a) and CD56 (PE, HCD56; both BioLegend) and intracellularly for IFN-γ (FITC, 25723.11, BD Biosciences) and analyzed by flow cytometry with the use of appropriate isotype controls. The gating strategy used is shown in Fig. S3. In some experiments, IL-12 was blocked during the coculture by addition of anti-human IL-12/IL-23 p40/70 antibody (C8.6, eBioscience).

### Cytokine Secretion Measurement by ELISA and Bead-based Immunoassay

Secretion of IL-12p70, IL-10, IFN-γ, IL-4 and IL-17 in the various experiments was quantified by CBA Flex Set (BD Biosciences), or by ELISA using a pretested antibody combination (OptEIA™ Set Human IFN-γ, BD Biosciences), according to the manufacturer’s instructions.

### Statistical Analysis

All results are presented in box-and-whisker plots, with boxes representing the lower quartile, the median and the upper quartile, while the whiskers show the lowest datum within 1.5 times the interquartile range of the lower quartile and the highest datum within 1.5 times the interquartile range of the upper quartile. Outliers not included between the whiskers are plotted with a circle. As interindividual values were not distributed normally, but all comparisons were between related samples, differences between groups were assessed using the Wilcoxon signed-rank test, calculated with PASW Statistics 19 (SPSS, an IBM Company, Chicago, IL, USA). p<0.05 was considered statistically significant (* in all figures), while p<0.01 is termed highly significant (** in all figures).

## Supporting Information

Figure S1 **Cytokine secretion patterns of TLR-3-DCs and cc-7-DCs.** DCs generated from peripheral blood of healthy donors were analyzed for their cytokine secretion patterns (n = 10). (A) Mature DCs were cocultured with CD40L expressing mouse fibroblasts for 24 hours, the concentration of IL-12p70 and IL-10 in the supernatants was measured by CBA, and the difference to the basal secretion of the same cell populations without CD40 ligation was calculated. (B) Comparison of the IL-12p70/IL-10 ratio for TLR-3-DCs and cc-7-DCs.
(TIF)

Figure S2 **Gating strategy used for phenotyping of DCs.** For the costimulatory profile of DCs shown in Figures 1 and 2, information on the surface molecules was gathered by flow cytometry. In order to exclude contaminating non-dendritic cells, a gate in the Forward Scatter/Side Scatter Plot was set before further analyses, as exemplified here for two different samples. The cells in the gate are assumed to be dendritic cells, the percentage of all recorded events is shown. One representative dot plot of (A) TLR-3-DCs and (B) cc-3-DCs prepared from peripheral blood of one healthy donor each is shown.
(TIF)

Figure S3 **Gating strategy used for intracellular IFN-γ staining of NK cells.** For intracellular IFN-γ staining as shown in Figure 6 A and C, cells from the coculture were surface stained for CD3 and CD56 and NK cells defined as CD3^−^CD56^+^ cells (A). Positivity for IFN-γ was measured by intracellular cytokine staining (B), with gates defined using appropriate isotype controls.
(TIF)
