# Supplementary figures and images for: CD86 and IL-12p70 Are Key Players for T Helper 1 Polarization and Natural Killer Cell Activation by Toll-Like Receptor-Induced Dendritic Cells
Source: PLoS One. 2012 Sep 4;7(9):e44266. doi: 10.1371/journal.pone.0044266 (PMC3433478; doi:10.1371/journal.pone.0044266)

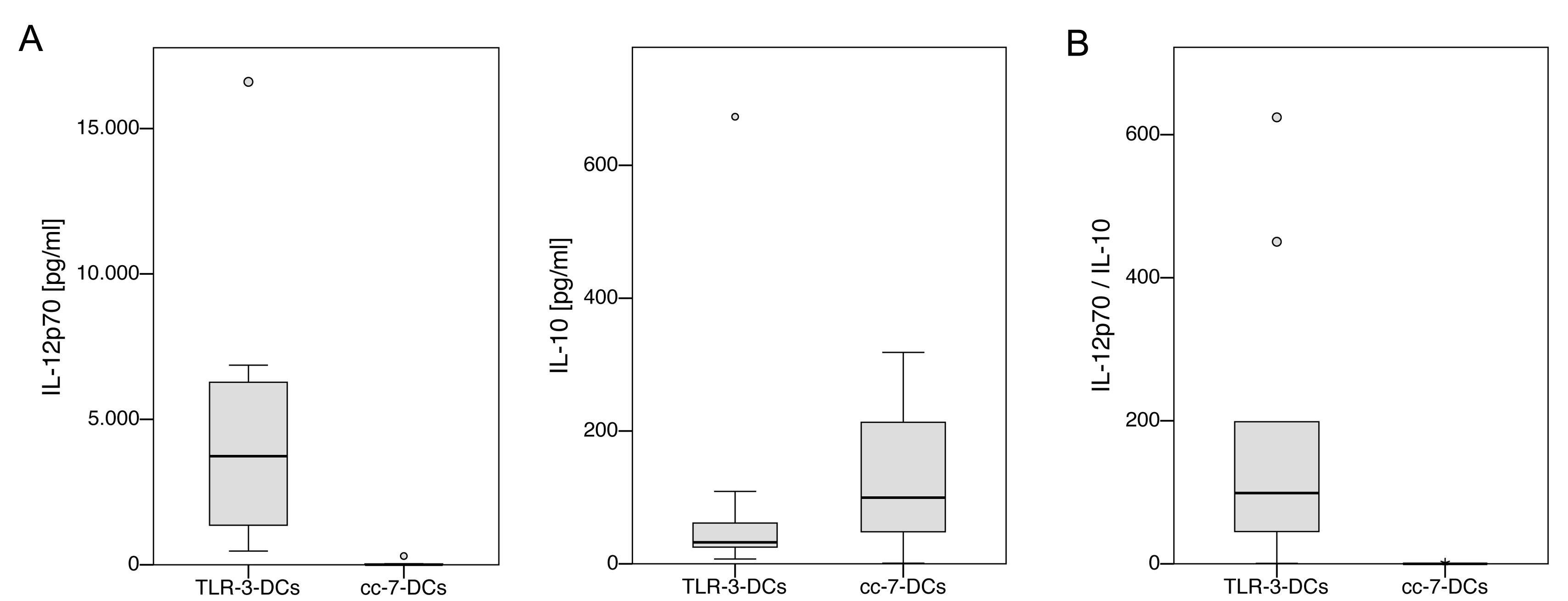

Supplement: Figure S1 — Cytokine secretion patterns of TLR-3-DCs and cc-7-DCs. DCs generated from peripheral blood of healthy donors were analyzed for their cytokine secretion patterns (n = 10). (A) Mature DCs were cocultured with CD40L expressing mouse fibroblasts for 24 hours, the concentration of IL-12p70 and IL-10 in the supernatants was measured by CBA, and the difference to the basal secretion of the same cell populations without CD40 ligation was calculated. (B) Comparison of the IL-12p70/IL-10 ratio for TLR-3-DCs and cc-7-DCs. (TIF) [file pone.0044266.s001.tif]

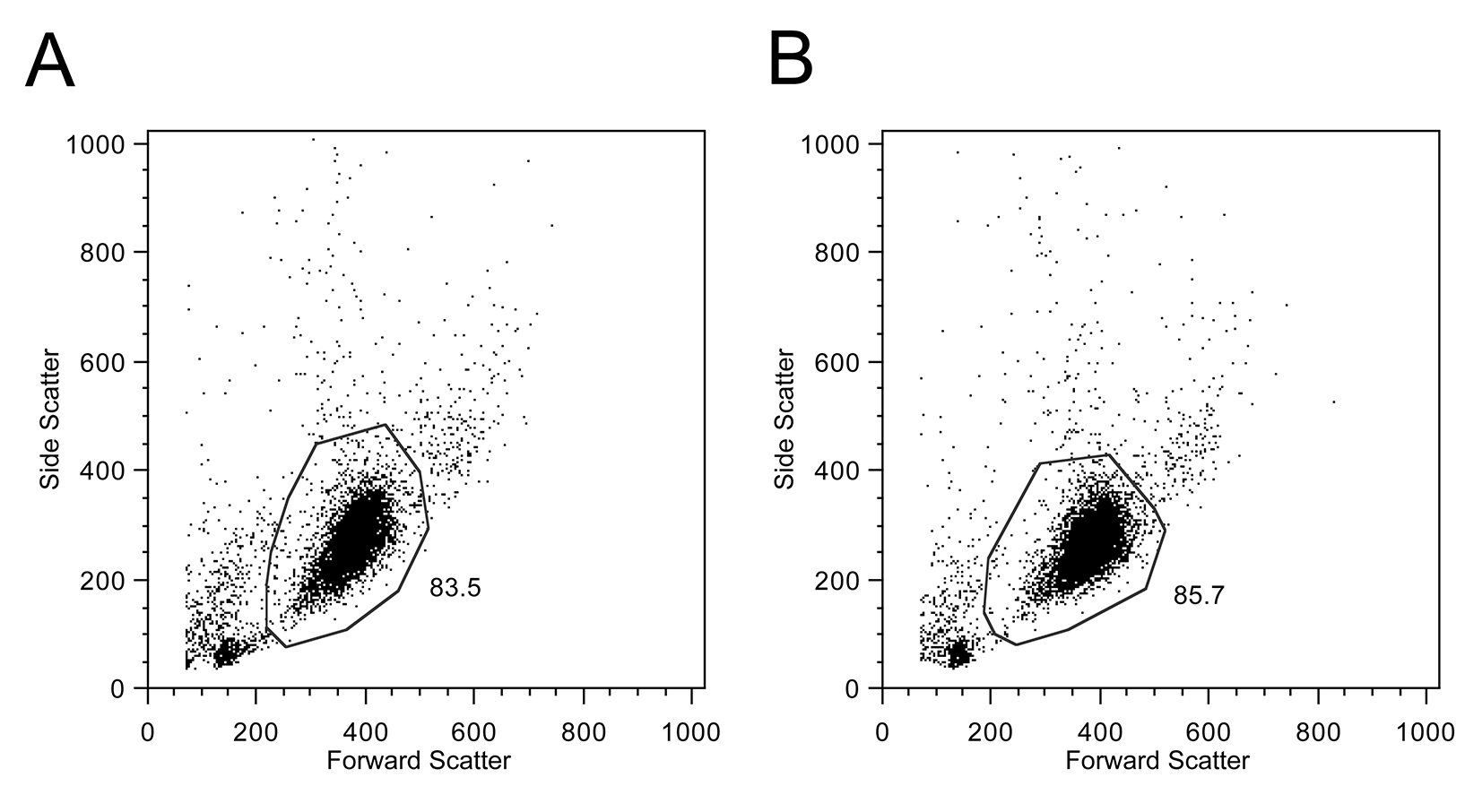

Supplement: Figure S2 — Gating strategy used for phenotyping of DCs. For the costimulatory profile of DCs shown in Figures 1 and 2, information on the surface molecules was gathered by flow cytometry. In order to exclude contaminating non-dendritic cells, a gate in the Forward Scatter/Side Scatter Plot was set before further analyses, as exemplified here for two different samples. The cells in the gate are assumed to be dendritic cells, the percentage of all recorded events is shown. One representative dot plot of (A) TLR-3-DCs and (B) cc-3-DCs prepared from peripheral blood of one healthy donor each is shown. (TIF) [file pone.0044266.s002.tif]

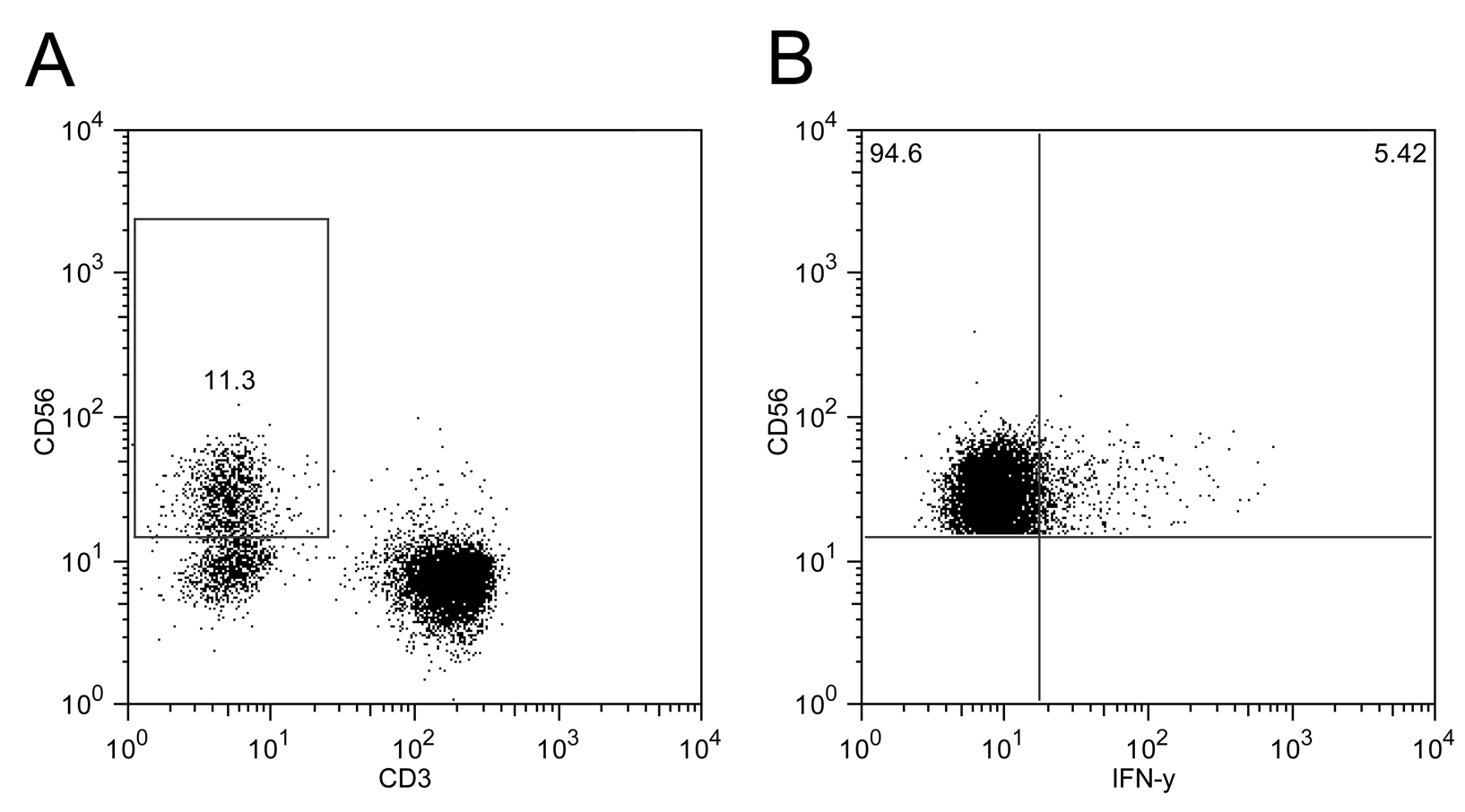

Supplement: Figure S3 — Gating strategy used for intracellular IFN-γ staining of NK cells. For intracellular IFN-γ staining as shown in Figure 6 A and C, cells from the coculture were surface stained for CD3 and CD56 and NK cells defined as CD3−CD56+ cells (A). Positivity for IFN-γ was measured by intracellular cytokine staining (B), with gates defined using appropriate isotype controls. (TIF) [file pone.0044266.s003.tif]
